# Exploring the plant lipidome: techniques, challenges, and prospects

**DOI:** 10.1007/s44307-024-00017-9

**Published:** 2024-03-11

**Authors:** Hao-Zhuo Liu, Yong-Kang Li, Yi-Li Chen, Ying Zhou, Sunil Kumar Sahu, Ningjing Liu, Hao Wu, Guanghou Shui, Qinfang Chen, Nan Yao

**Affiliations:** 1 State Key Laboratory of Biocontrol, Guangdong Provincial Key Laboratory of Plant Resources, School of Life Sciences, Sun Yat-Sen University, Guangzhou, 510275 China; 2 State Key Laboratory of Agricultural Genomics, Key Laboratory of Genomics, Ministry of Agriculture, BGI Research, Shenzhen, 518083 China; 3 School of Life Sciences, East China Normal University, Shanghai, 200241 China; 4 State Key Laboratory of Molecular Developmental Biology, Institute of Genetics and Developmental Biology, Chinese Academy of Sciences, Beijing, 100101 China

**Keywords:** Lipidomics, Plant lipids, Biomolecules, Mass spectrometry, Environmental stress

## Abstract

Plant lipids are a diverse group of biomolecules that play essential roles in plant architecture, physiology, and signaling. To advance our understanding of plant biology and facilitate innovations in plant-based product development, we must have precise methods for the comprehensive analysis of plant lipids. Here, we present a comprehensive overview of current research investigating plant lipids, including their structures, metabolism, and functions. We explore major lipid classes, i.e. fatty acids, glyceroglycolipids, glycerophospholipids, sphingolipids, and phytosterols, and discuss their subcellular distributions. Furthermore, we emphasize the significance of lipidomics research techniques, particularly chromatography-mass spectrometry, for accurate lipid analysis. Special attention is given to lipids as crucial signal receptors and signaling molecules that influence plant growth and responses to environmental challenges. We address research challenges in lipidomics, such as in identifying and quantifying lipids, separating isomers, and avoiding batch effects and ion suppression. Finally, we delve into the practical applications of lipidomics, including its integration with other omics methodologies, lipid visualization, and innovative analytical approaches. This review thus provides valuable insights into the field of plant lipidomics and its potential contributions to plant biology.

## Introduction

Lipids are small hydrophobic or amphipathic molecules that are readily soluble in organic solvents. These molecules are vital components of plant cells that play crucial roles in many biological processes. Plants have thousands of types of lipid molecules, which can be divided into fatty acids, glycerolipids, sphingolipids, and sterols based on their structures and chemical properties. Some lipids participate in the cell membrane system as membrane lipids (Horvath and Daum 2013; Kobayashi et al. 2016), some participate in metabolism and the generation of energy (Fan et al. 2019), and others regulate biological activities by functioning as signaling molecules (Bi et al. 2014; Huang et al. 2021; Zeng and Yao 2022). Moreover, the lipid layer on the plant epidermis serves as a basic protective system for plants (Riederer and Schreiber 2001).

**Fig. 1:**
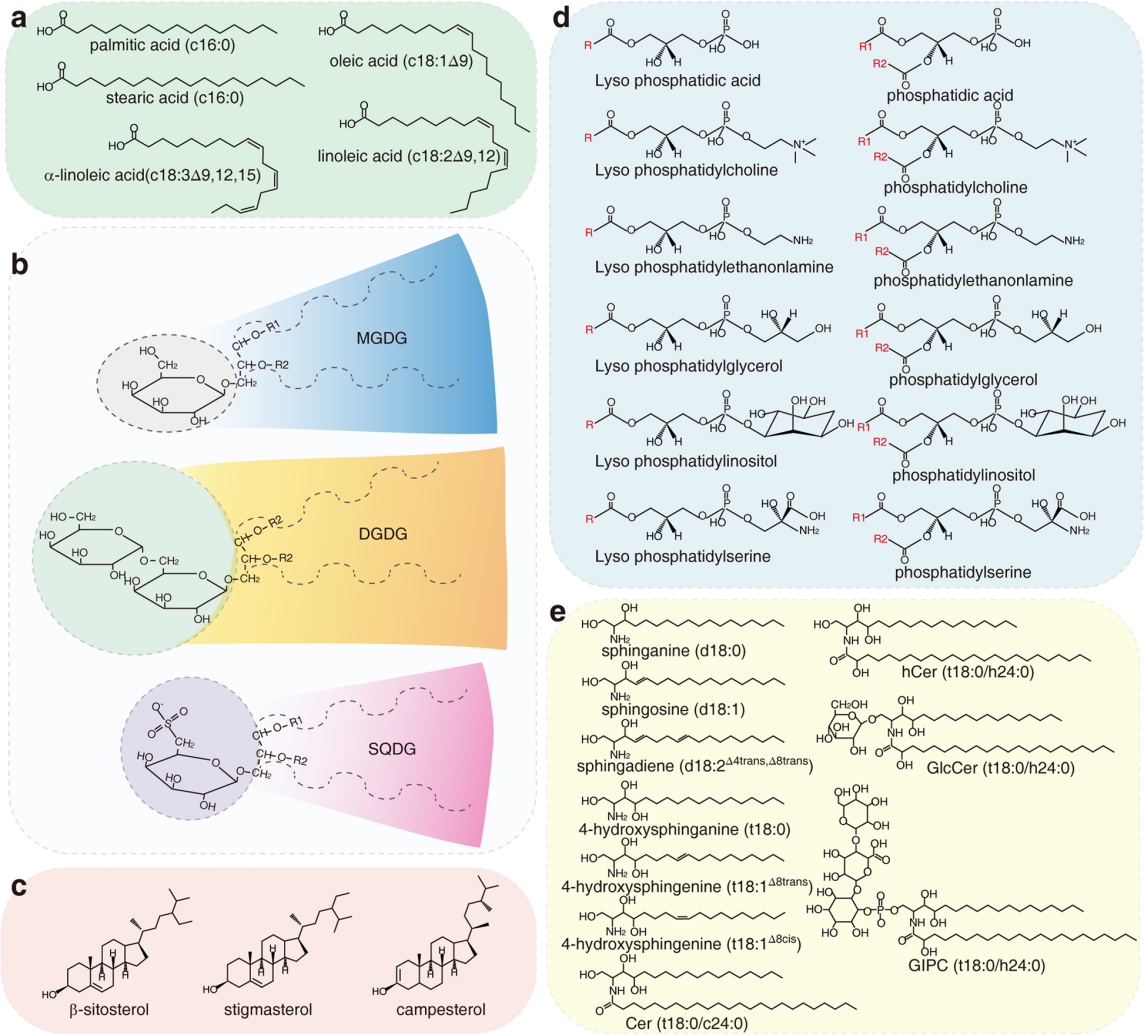
Structures of major functional lipids. **a** Chemical structures of common fatty acids, showing the hydrocarbon chain and terminal carboxylic group. **b** Examples of glyceroglycolipid categories. Glyceroglycolipids are neutral lipids consisting of a diacylglycerol and a hexose group. MGDG, monogalactosyl diacylglycerol, DGDG, digalactosyl diacylglycerol, SQDG, sulfoquinovosyl diacylglycerol. **c** Phytosterol consists of a tetracyclic ring backbone, with a hydroxyl group at the 3rd carbon atom and an aliphatic side chain containing eight to ten carbon atoms at the 17th carbon atom. **d** A phospholipid molecule consists of a polar phosphate head, which is hydrophilic, and a non-polar, hydrophobic lipid tail. **e** The chemical structures of representative sphingolipids. Sphingolipids consist of a sphingoid long-chain base, a fatty acid chain, and a head group

Fatty acids (FAs) are carboxylic acids consisting of a hydrocarbon chain and a terminal carboxylic group (Fig. 1) and are mainly produced in plastids. Most FAs exist as glycerolipids or sphingolipids, and other extracellular lipids (cutin and waxes), and only a few exist in the free state (Lim et al. 2017). Glyceroglycolipids are neutral lipids consisting of a diacylglycerol and a hexose. The most common glyceroglycolipids in plants include monogalactosyl diacylglycerol (MGDG), digalactosyl diacylglycerol (DGDG), and sulfoquinovosyl diacylglycerol (SQDG). Phytosterols are isoprenoid derivatives belonging to the triterpenoid family (Fig. 1). Similar to animal sterols, phytosterols contain a tetracyclic ring backbone with a hydroxyl group at the 3rd carbon atom and an aliphatic side chain containing eight to ten carbon atoms at the 17th carbon atom (Valitova et al. 2016).

Glycerophospholipids, which are also referred to as phospholipids, consist of a diacylglycerol and a polar phosphate group. The lipophilicity of glycerol and the hydrophilicity of the polar head group make phospholipids amphiphilic, allowing them to easily form a bilayer structure, with FA chains toward the inside and polar groups toward the outside. Phospholipids are characterized as phosphatidic acids (PAs), phosphatidylcholines (PCs), phosphatidylethanolamines (PEs), phosphatidylglycerols, (PGs), phosphatidylinositols (PIs), and phosphatidylserines (PSs) based on their polar groups.

Sphingolipids consist of a sphingoid long-chain base (LCB), an FA chain, and a head group. Based on the head group and FA chain, sphingolipids are characterized as glycosyl inositolphosphoceramides (GIPCs), glucosylceramides (GlcCers), ceramides, hydroxyceramides (hCers), and LCBs, or their phosphate derivatives (Fig. 1). In Arabidopsis (*Arabidopsis thaliana*), GIPCs account for 60–65% of total lipids, GlcCers account for 30% of total lipids, and the others make up the rest (Markham et al. 2006).

In the 1950s, thin-layer chromatography (TLC) was the main method used for analyzing lipids. TLC is inexpensive and simple and is still widely used in many laboratories today. Although TLC can be performed easily and quickly for lipid analysis, the accurate identification and quantification of lipids requires advanced techniques. Chromatography-mass spectrometry (MS) provides a platform for the accurate analysis of lipids. For low molecular weight lipids such as FAs and sterols, gas chromatography (GC) electron-impact MS is the most common technique used for analysis (Christie et al. 2007). This method was initially used for the separation and identification of lipid molecules in high ionization energy mode. It can also be used to distinguish the isomers of double-bonded lipids in low ionization energy mode after optimization (Hejazi et al. 2009b). Moreover, field ionization (FI) can be combined with orthogonal acceleration time-of-flight mass spectrometry (TOF–MS) and GC × GC to analyze the structures of lipids more accurately, even to detect new structures (Hejazi et al. 2009a).

Since most lipids require derivatization in gas phase mode prior to analysis, sample preparation is a relatively complicated process. Incomplete derivatization commonly occurs, leading to quantitative and qualitative inaccuracies (Beale et al. 2018). Currently, the most commonly used strategy in lipidomics analysis is ionization prior to MS scanning. The major methods include shotgun and other approaches based on liquid chromatography-mass spectrometry (LC–MS). With the development of ionization techniques and MS, various types of ionization can be utilized in lipidomics, such as electrospray ionization (ESI), matrix-assisted laser desorption/ionization (MALDI), atmospheric pressure chemical ionization (APCI), secondary ion mass spectrometry (SIMS), and desorption ESI (DESI). The analysis methods for tandem MS include product ion scan, precursor-ion scan, neutral-loss scan, selected reaction monitoring (SRM), multiple reaction monitoring (MRM), and others (Yang and Han 2016). Using different tandem ionization techniques and MS scan mode, we can achieve most of the requirements for lipidomics. DESI was recently used as the ionization mode to increase the resolution of shotgun lipidomics. The rapid, simple, high-throughput nature of the shotgun approach makes it the major method in untargeted lipidomics. Ultra-high-performance liquid chromatography (UHPLC) has gradually replaced HPLC in recent years, significantly enhancing the resolution of lipid identification (Zullig and Kofeler 2021).

## The functions of lipids

### Functions of lipids in organelles

Lipids can easily form continuous hydrophobic barriers due to their hydrophobic properties. These hydrophobic barriers constitute the main area of biological membranes and these membranes divide the eukaryotic cell into various independent and functionally related subregions. Phospholipids are the basic units in plant lipid membranes, which provide support for other lipids, proteins, and polysaccharides. Lipids do not randomly exist in membranes; their specific distribution affects the structures, functions, and properties of membranes. Therefore, identifying the lipid components in different membranes is important for unveiling the different functional subregions of plant cells.

The plant epidermis is covered with a continuous transparent hydrophobic layer that regulates the exchange of water and gas between the plant and the external environment and protects the plant against biotic and abiotic stress (Delude et al. 2016). This hydrophobic cover on the plant surface contains large amounts of lipids, which attach to the outside of the cell wall to form the inner cuticle, surface waxy mixed layer, and epidermal waxy outer layer (Samuels et al. 2008). Cutins are a class of lipid polymers primarily formed by long-chain FAs. Hexadecane and octadecane fatty acids are catalyzed by cytochrome P450 to form dicarboxylic acids, which are further converted to monoacylglycerol (MAG) by glycerol-3-phosphate acyl-CoA transferases (GPATs) (Yang et al. 2012). Cutin Deficient 1 (CD1), a GDSL family protein discovered in tomato (*Solanum lycopersicum*), catalyzes the polyesterification of MAGs in vitro to produce linear cutin oligomers (Yeats et al. 2014), but the pathway that forms cutin polymers remains unclear. In general, cutin forms the backbone that supports substances on the plant surface and protects plants from mechanical damage. However, waxiness plays a vital role in water conservation and plant–environment interactions (such as plant–insect interactions) (Riederer and Schreiber 2001).

The mitochondrion is the main organelle that produces energy in cells, whereas the chloroplast is the site of photosynthesis in plants. Both organelles have a double-layer membrane. In plant mitochondria, lipids are not symmetrically distributed in the inner or outer membrane and matrix, but phospholipids are the predominant FAs in these regions (PC and PE roughly account for 80% of total lipids in mitochondria) (Horvath and Daum 2013). The phospholipid cardiolipin (CL), an essential component of the respiratory chain (Gohil et al. 2004), can be synthesized from PGs and diacylglycerol (DAG) in mitochondria (Liu et al. 2023b). Sphingolipids also are present in mitochondria and play vital roles in mitochondria-associated cell death (Bi et al. 2014). Interesting, Liu et al. (2023b) observed the accumulation of GIPCs in leaf mitochondria and suggested that GIPC might function in establishing communication between mitochondria and other organelles.

Apart from their double-layer membrane structure, chloroplasts have also evolved thylakoid membranes. The chloroplast-associated nonspecific phospholipase C6 promotes glycerolipid turnover in the membrane, leading to the accumulation of TAG in oil seeds (Cai et al. 2020). The sphingolipidome of chloroplasts differed from that of other membrane components in total leaf samples, with higher concentrations of free long-chain bases and hydroxyceramides and a greater proportion of complex sphingolipids with 16C FAs (Yang et al. 2024). The chloroplast membrane can affect embryogenesis and germination by enhancing photosynthetic efficiency (Fujii et al. 2014; Cook et al. 2021). More specifically, MGDG and DGDG make up 60–80% of total lipids in the chloroplast membrane, while the remaining lipids primarily consist of SQDG, PG, and trace amounts of PI (Kobayashi et al. 2016). The distribution of lipids in the chloroplast is closely related to chloroplast function (Cook et al. 2021).

Since the plasma membrane functions as a cellular barrier, it encounters external biotic or abiotic stress and provides a relatively stable environment, allowing the cell to perform various biological functions. The dominant types of lipids in the plasma membrane are phospholipids, sphingolipids, and sterols. Similar to most membranes based on a phospholipid backbone, the major phospholipids in the plasma membrane are PC and PE, which contain long-chain fatty acids, whereas the contents of PG, PI, PA, and PS are relatively low. As the first gateway for the intercellular exchange of materials and signal transmission, the plasma membrane is rich in polyphosphorylated PIPs, which can act as signaling molecules. The plasma membrane is also rich in sphingolipids (comprising ~ 40–50% of total membrane lipids), primarily GIPCs (~ 70%) (Grison et al. 2015). The head group of negatively charged GIPCs plays a vital role in forming and maintaining stable differences in membrane potential. Free sterols constitute the major sterol lipid class (∼80%) in the plasma membrane, whereas conjugated sterols are present at lower levels (Grison et al. 2015). An evolving model describes how membranes are occupied by fluctuating nanoscale assemblies of sphingolipids, sterols, and proteins that can be stabilized into platforms that are important for signaling, viral infection, and membrane trafficking (Simons and Gerl 2010; Chen et al. 2023).

The endoplasmic reticulum (ER), an organelle enclosed by a continuous membrane system made of double lipid layers, is an extraordinarily active site for the biosynthesis of materials such as lipids, saccharides, and various protective compounds (Sparkes et al. 2009). The ER membrane provides a relatively stable micro-region for substance metabolism and supports most trans-membrane enzymes (Kanehara et al. 2022). Furthermore, the ER membrane is in a relatively active, dynamic state and can convey the synthesized substance to different locations of the cell through its extensive network system (Sparkes et al. 2009).

The Golgi is a complex formed by a flat sac-like membrane and surrounding vesicles. This organelle is usually located between the ER and plasma membrane and is the main site for intracellular material processing (including protein glycosylation modification and lipid processing) and material sorting (Dupree and Sherrier 1998). From the perspective of lipid composition, the dominant components of the ER membrane and Golgi membrane are phospholipids, sterols, and sphingolipids. The phospholipids primarily include PCs and PEs, the sterols are mainly free sterols, and the sphingolipids are mainly glucosylceramides (Fouillen et al. 2018). In plants, the proportion of various lipids in the internal membrane system is relatively stable, and the membrane structure will collapse if an imbalance in the proportion of lipids occurs. For instance, the accumulation of PEs and PCs will lead to unusual ER membrane extensions (Eastmond et al. 2010).

Lipid droplets (LDs), which exist in various organisms, contain a hydrophobic core surrounded by a phospholipid monolayer (Xu et al. 2023). LDs play vital roles in seedling development, pollen formation, and abiotic stress responses (Ischebeck et al. 2020). Although the size and number of LDs vary in different cells (Xu et al. 2023), all LDs contain large amounts of neutral lipids, specifically storage lipids (such as TAG and steryl esters). These neutral lipids can be synthesized de novo from the ER membrane and can also form from redundant structural membrane lipids or other lipids via lipotransferase (Zhang et al. 2009). Vast amounts of neutral lipids can aggregate into the convex point in the ER lumen. When the convex point reaches a certain size, it is released into the plasma to form simple LDs covered by a phospholipid monolayer. The hyper-accumulation of most lipids in plants, such as FAs, PAs, ceramides and sterols, can cause significant harm to cells. Storage lipids, which function as energy substances in the cell, are one of the main sources of metabolic energy production from lipids. Through the process of lipophagy, TAGs in LDs can be degraded into free FAs, which can enter mitochondria or peroxisomes to produce energy for various cellular activities (Fan et al. 2019). These storage lipids in plants, especially TAGs, are the main components of lipids in seed oils. It is important to increase the accumulation of TAGs and other storage lipids in oilseed crops.

### The functions of lipids as signaling molecules

Recent research indicates that lipids are not only the basic units of the membrane system but also the primary receptors of intracellular and extracellular signaling substances, responding to and transmitting these signals. The plasma membrane of Arabidopsis contains large amounts of GIPCs, which can combine with Na^+^ and regulate Ca^2+^ channels in the plasma membrane, functioning as receptors for salt-stress signals (Jiang et al. 2019). GIPCs can also function as receptors to identify Necrosis and Ethylene-inducing Peptide 1-like (NLP) proteins, a class of toxic proteins from pathogens, in plant–pathogen interactions (Lenarcic et al. 2017).

Several types of free lipids, such as free FAs, phosphatidic acids, sphingosines, and ceramides, act as signaling molecules to regulate various biological processes in plants. Free FAs interact with plant hormones during growth, development, and responses to external stress. Some oxidized forms of unsaturated FAs (such as arachidonic acid, linoleic acid, and alpha-linolenic acid) can act as signaling molecules to activate most physiological responses in vivo. For example, the oxidized form of alpha-linolenic acid is a precursor of jasmonate (JA) (Tang et al. 2024), thereby functioning in the JA-mediated pathway, and affects the formation of plant cell walls and protective responses to insects. Moreover, the C18:2 and C18:3 linoleic acids in plants directly affect the plant’s ability to defend against pathogens (Ongena et al. 2004).

Glycerophospholipids (such as PAs, PIs, and their phosphorylated forms) are thought to function as signaling molecules. PAs function as crucial secondary messengers during plant responses to external stimuli and stress. When plants are infected with pathogens, large amounts of PAs accumulate to increase reactive oxygen species (ROS) production and the hypersensitive response (Laxalt and Munnik 2002). PIs also serve as critical signaling molecules, which not only interact with proteins to activate their functions but can also be degraded to the form the soluble signaling molecules inositol polyphosphates (IPPs) via the activity of PI-phospholipase C (PI-PLC) (Gunesekera et al. 2007).

Sphingolipids, including free sphingosines (long chain bases, LCBs), ceramides, and their phosphorylated forms, are vital signaling molecules (Bi et al. 2014; Zeng and Yao 2022). LCBs/LCB-Ps and ceramides/ceramide-1-Ps function as pairs of signaling molecules responsible for programmed cell death. The LCB component 4-hydroxysphinganine activates ROS to upregulate resistance-related genes and causes plant cell death to strengthen the plant’s defensive ability against pathogens (Peer et al. 2010; Liu et al. 2020b; Zeng et al. 2021; Zeng and Yao 2022). A recent study showed that the JA-mediated pathway also affects the metabolism of ceramides. For instance, JA induces the accumulation of ceramides and hydroxyceramides and is involved in regulating the transcription of ceramide-related genes. The deficiency of sphingolipid-related enzymes impairs the defensive function of JA against insects (Huang et al. 2021 & 2022).

## Analysis of plant lipids: mass spectrometry platforms

The lipidome (the total contents of molecular species of each lipid class) is analyzed using lipidomics techniques. Although many lipidomic approaches may not fully uncover the subtleties of different molecular species of lipids, such as the complete FA compositions of certain lipid classes (Romsdahl et al. 2022), several LC–MS methods have been successfully employed to characterize and quantify the lipidome (Herrfurth et al. 2021; Kehelpannala et al. 2021). Here, we describe the entire lipidomics workflow and highlight the crucial points.

### Key elements of the mass spectrometry platform to analyze plant lipids

MS-based lipidomics is rapid compared to traditional lipid analysis techniques such as TLC. Because MS measurements are based on mass-to-charge ratios (*m/z*), ionization is essential for this process. Ionization causes sample components to become positively or negatively charged. The ion-source parameters and their optimum values depend on the specific electrospray ionization source applied. For example, in some ionization source designs, the optimum voltage difference between the needle and counter-electrode is approximately 4.5–5 kV, while the optimum voltage is approximately 3 kV in other designs. Most commonly used systems allow for automatic optimization of the parameters for the ion source and mass spectrometer. These procedures search for optimum ion production and transmission toward the analyzer.

Mass analyzers (also known as separators) are another basic component of mass spectrometers. After ionization, the ions are accelerated into mass analyzers for separation. Various types of field mass spectrometers are employed, which use magnetic sectors or additional electrostatic sectors, such as quadrupole mass spectrometers, time-of-flight mass spectrometers (TOF–MS), or ion trap mass spectrometers. To achieve fragmentation, multiple analyzers are combined (MS/MS mass spectrometry). The major types of mass spectrometers that are currently used for lipidomic analysis are triple quadrupole (QqQ), quadrupole TOF (Q-TOF), Q-linear ion trap (Q-LIT), and LIT-Fourier transform ion cyclotron resonance (LIT-FT-ICR) (Wu et al. 2020). The modular instrumentation strategy promotes the combination of various sample introduction systems, ion sources, analyzers, and detectors. This approach provides a high degree of flexibility in instrumentation to effectively address the challenges encountered in lipidomics. The performance of various MS modules may vary, which can be observed in terms of sample stability, detection sensitivity, and other factors. Users can select the most suitable experimental scheme based on the reported experimental results or optimize the detection method according to their specific experimental requirements and instrument conditions.

The most commonly used MS-based lipidomic approach involves the analysis of transitions of precursor *m/z* and fragment *m/z* to identify and quantify lipid species. This analysis is efficiently performed using a triple quadrupole mass spectrometer. Plant sphingolipids, in particular, are amenable to analysis by electrospray ionization triple quadrupole mass spectrometry coupled with liquid chromatography. A QqQ mass spectrometer contains two analyzer quadrupoles (Q1 and Q3) with a collision cell (the second quadrupole, Q2) in between. Q1 passes precursor ions to the collision cell, where the ions are fragmented and passed to Q2; Q3 passes fragment ions to the detector. The structures of sphingolipids can be analyzed by precursor ion scanning and product ion scanning to explore structural variations. In a study using the TurboIonSpray source of a 4000 QTRAP LC/MS/MS System, sphingolipids were detected with a needle temperature of 100°C, needle voltage + 5000 V, curtain gas at 10 psi, nebulizing gas (GS1) at 20 psi, focusing gas (GS2) at 0 psi, and the interface heater engaged. Declustering potential and collision energy were optimized on a compound-dependent basis (Markham and Jaworski 2007).

### Fragmentation patterns of plant lipids

There are many types of MS instruments with different ionization sources. Depending on the ionization method, the molecule may break apart into a population of smaller fragments. Below, we summarize some general rules on how plant lipids with various chemical skeletons could be ionized.

**Fig. 2:**
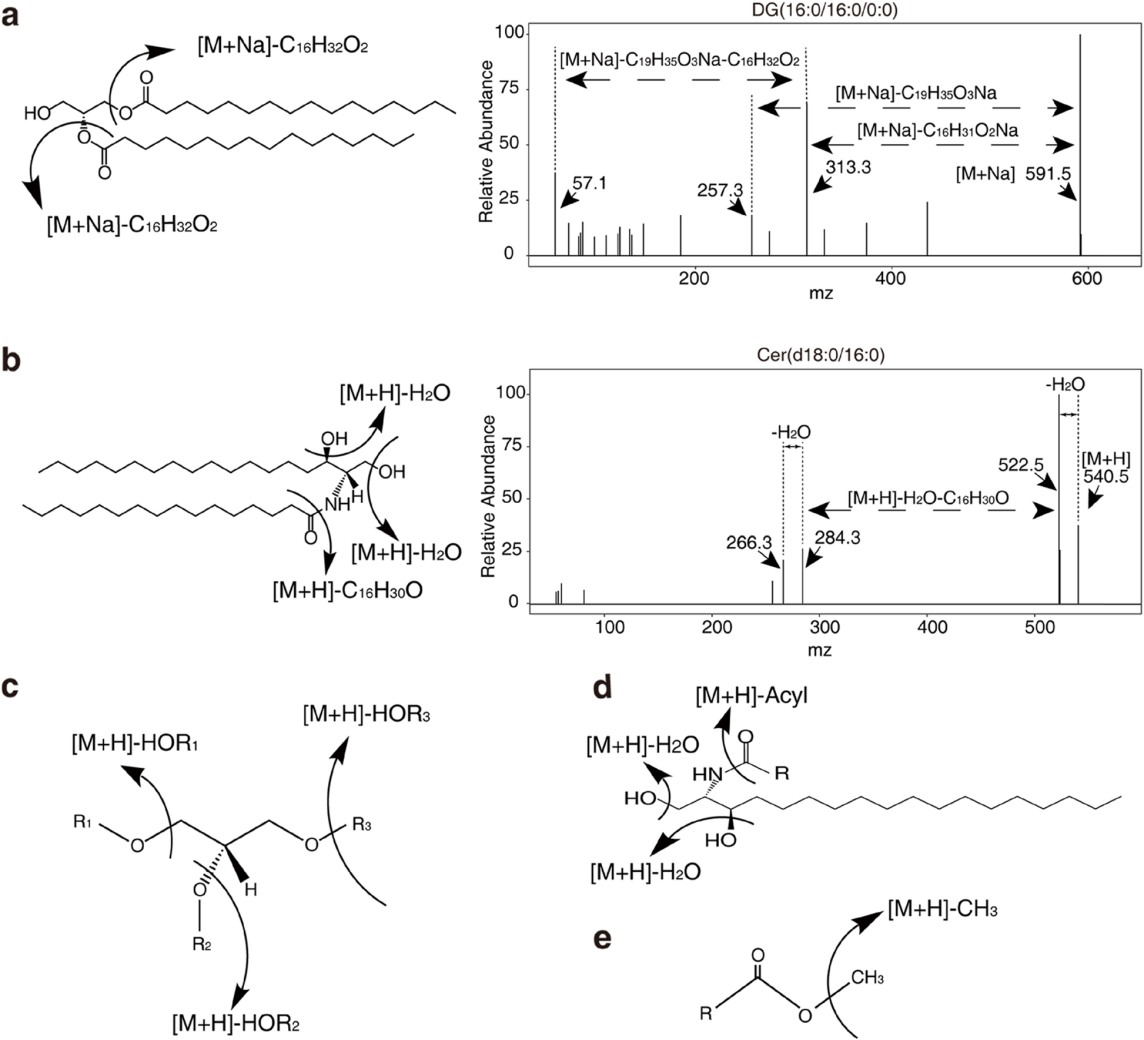
Validated fragmentation rules of various lipids. **a** Product-ion mass spectra of glyceroglycolipid DG (16:0/16:0/0:0) at 40V voltage in positive ion mode. Three types of smaller molecules could be produced, C_16_H_31_O_2_Na, C_19_H_35_O_3_Na, and C_16_H_32_O_2_, because Na^+^ could be connected at different positions in the original molecule. **b** Mapping the fragmentation of ceramide (d18:0/16:0) at 40V voltage in positive ion mode. The resulting spectral information indicates that three groups of fragments could be produced: two -OH and one C_16_H_30_O. **c**-**e** Typical fragmentation patterns of glycerides (**c**), sphingolipids (**d**), and methylated fatty acids (**e**). The one-way arrows indicate where fragmentation of the lipid molecule occurs

The hydroxyl hydrogen atoms on the basic skeleton of glycerides can be replaced by different groups. The glycerides in plants are mainly composed of acylglycerol, glyceroglycolipids, and glycerophospholipids. The detection of glycerides is generally performed by LC–MS. Under actual experimental conditions, when fragments of the glyceroglycolipid DG (16:0/16:0/0:0) were collected at 40 V in positive ion mode, the resulting spectral information showed that its parent ion was an adduct containing Na^+^. Because Na^+^ could be connected at different positions, three types of smaller molecules could be produced: C_16_H_31_O_2_Na, C_19_H_35_O_3_Na, and C_16_H_32_O_2_. Hence, the fragmentation was successful (Fig. 2a). Most glycerides can be separated using a reverse-phase chromatography column such as a C18 column. The general fragmentation rule is shown in Fig. 2c.

Sphingolipids can be detected by LC–MS and are usually separated through a reverse phase chromatography column (e.g., a C8 or C18 column). Sphingolipids are a diverse class of compounds containing multiple LCBs and fatty acids with varying degrees of saturation and hydroxylation. Fatty acids usually differ by 2 carbon units with an *m/z* of 28; hence, sphingolipids were identified in each sample as a group of compounds that differed by an *m/z* of 28 (Pata et al. 2010). In actual circumstances, when fragments of ceramide (d18:0/16:0) were acquired at 40 V in positive ion mode, the resulting spectral information revealed three groups of molecules that could be detected, including two -OH and a C_16_H_30_O, indicating that fragmentation was successful (Fig. 2b). The general fragmentation rule is shown in Fig. 2d. In each case, fractions containing the majority of charged sphingolipids (usually [M + H]^+^) are infused into the mass spectrometer, and a profile typical for sphingolipids is produced.

Fatty acids are generally detected by gas chromatography-mass spectrometry (GC–MS). Chemical derivatization is often introduced in MS-based analysis due to the inherent poor ionization efficiency and selectivity of fatty acids and fatty acyls. The formation of alkyl esters (methyl, ethyl, propyl, or butyl esters) is the most common type of derivative reaction used in the analysis of fatty acids (Watkins 2020). The MS fragmentation rule for widely analyzed methyl esters is shown in Fig. 2e.

Plant sterols are cholesterol-like terpenoids that are widespread in the kingdom Plantae. APCI-based LC–MS has been widely utilized to analyze plant sterols due to its ionization efficiency without the need for derivatization, primarily forming [M + H–H_2_O]^+^ ions (Mo et al. 2013; Gu et al. 2016). Despite its widespread use, the chemical structures of many complex plant sterols still need to be fully characterized (Evtyugin et al. 2023). Although many analytical strategies can be employed to analyze sterols, the molecules may fragment into different components using various derivatizing agents and ionization sources (Gachumi and El-Aneed 2017). For more detailed information on plant sterol fragmentation rules, please see literatures (Wewer et al. 2011; Gachumi and EI-Aneed 2017; Evtyugin et al. 2023).

### Sample preparation for LC–MS analysis

**Fig. 3:**
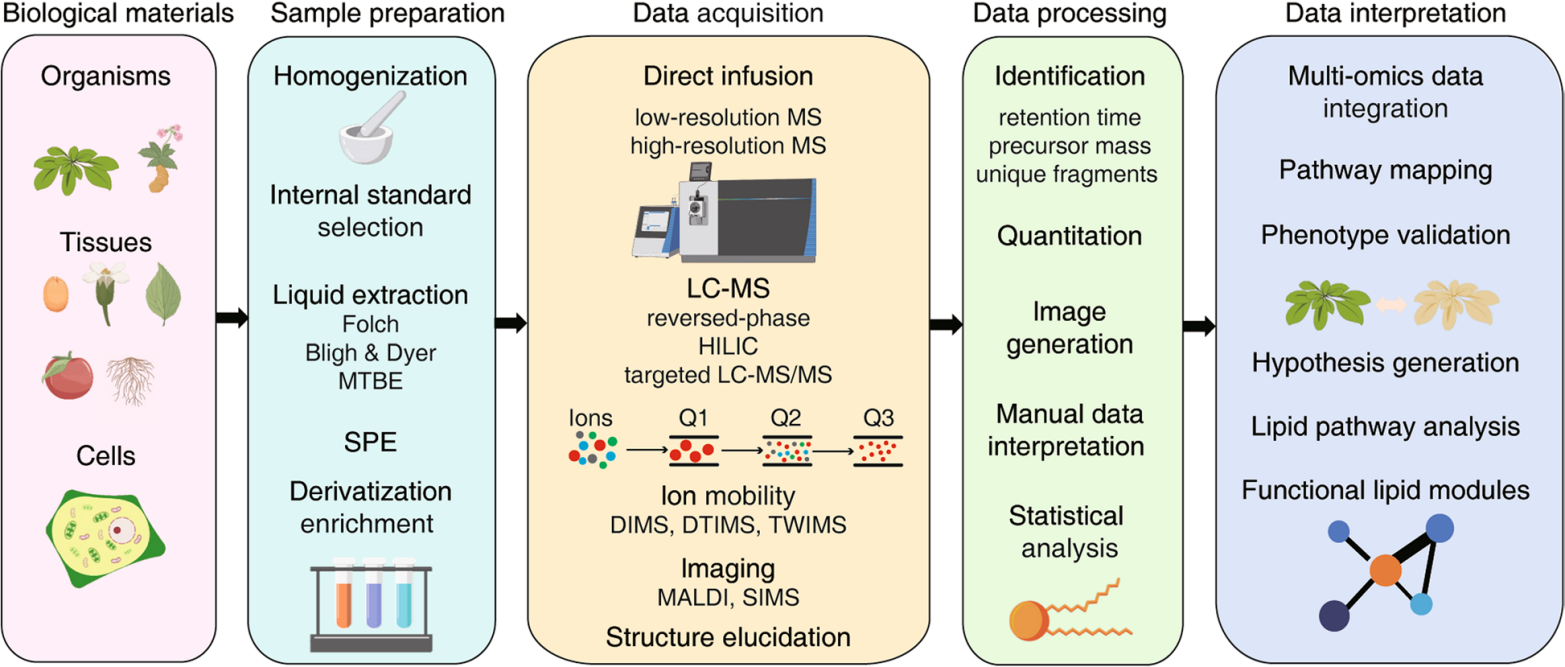
The lipidomics workflow. Lipidomics involves collecting samples of various biological materials, such as whole organisms, different tissues, or cells, and extracting their lipids. The lipid extracts are then combined with the appropriate internal standards for semi- or relative-quantification of the biological lipidomes, primarily using mass spectrometry (MS). Targeted or non-targeted approaches can be used in quantitative MS-based lipidomics. The use of MS-imaging to map the spatial distribution of different lipids in tissue sections is also increasing. After the lipidome dataset is obtained, phenotypic validation and pathway mapping can be performed using various bioinformatics techniques

Proper sample preparation is a decisive factor for a successful experiment. Here, we introduce sample preparation methods that are used when analyzing plant lipids by LC–MS. All sample preparation methods can be divided into three steps: sample collection, preservation, and extraction (Fig. 3).

For sample collection, a reasonable sampling standard must comply with the aims of the experiment and the specific experimental objective. When analyzing the lipid contents of roots, stems, leaves, and other plant organs, the amount of plant tissues that should be collected is determined by the actual growth state of the plant: different amounts of sample and different pretreatment methods are chosen for young leaf tissues with higher water content and highly lignified roots. With the development of ion sources and ion optics in mass spectrometers, greater sensitivity in MS analysis has been achieved. The current instrument detection limit (IDL) of MS, i.e., the analyte concentration required to produce a signal that is distinguishable from the noise level within a particular statistical confidence limit, could be pg or even fg, and therefore, only a small amount of sample is sufficient for detecting lipids in plants. In most experiments, the fresh weight of each biological replicate is approximately 100–500 mg, and the weight should be increased for samples with high water content. The sample should be extracted as soon as possible to ensure the minimum degradation of lipids during sample preservation. If continuous sample collection is required over a period of time, the sample could be sealed and stored at –80°C for roughly six months to one year without generating extreme lipid degradation. Nevertheless, if unprocessed or processed samples are stored at − 80°C for a long period of time, the stability of the lipid species to be examined should be verified.

In lipidomics, any extraction method serves two primary purposes. First, it simplifies the sample by eliminating unwanted nonlipid compounds. Second, it increases the concentrations of the analytes of interest (in this case, lipids), resulting in improved signal-to-noise ratios. The most commonly used sample preparation technique in lipidomics is liquid–liquid extraction, which relies on biphasic chloroform–methanol-water mixtures (Lebaron and Folch 1959; Bligh and Dyer 1959). Using this technique, the lipids are found in the organic chloroform/methanol phase, while the aqueous phase contains the more hydrophilic compounds and salts. Lipidomic extraction methods are constantly being modified to meet different experimental needs, such as reducing degradation and improve solubility, resulting in increased extraction efficiency (Guazzotti et al. 2023; Ryan et al. 2023). Below, we describe specialized extraction methods for phospholipids, galactolipids, and sphingolipids.

#### Preparing plant phospholipids and galactolipids for analysis

Total plant lipids are generally extracted using previously established methods (Welti et al*.* 2002; Devaiah et al. 2006; Zhou et al. 2022a). Here, we describe the key steps in detail.

1. Organs are harvested and transferred to 3 ml of 75°C isopropanol with 0.01% butylated hydroxytoluene (BHT) in a 50 mL glass tube with a Teflon-lined screw cap to fully immerse the tissue. The sample is heated at 75°C for 15 min.
2. After adding 1.5 mL of CHCl_3_ and 0.6 mL of water to each sample, the tubes are vortexed and shaken at 100 rpm at room temperature for 1 h. Each lipid extract is transferred to a new 50 mL tube, and the tissues are re-extracted with 4 mL CHCl_3_: MeOH (2:1 v/v) with 0.01% BHT. The tubes are shaken for 30 min. The solvents from the tissues are removed, transferred to 50 mL tubes, and combined with the lipid extracts. This extraction procedure is repeated three times, until all of the remaining plant tissue appears white.
3. The combined extracts are washed with 1 ml of 1 M KCl, vortexed, and centrifuged at 500 × *g* for 10 min. The upper phase is discarded, and 2 mL water is added to the combined extracts. The samples are vortexed, centrifuged at 500 × *g* for 10 min, and the upper phase discarded. The extracts are evaporated under nitrogen, and the lipid extract is dissolved in 1 mL of CHCl_3_.
4. The remaining plant tissue is heated overnight at 105°C and weighed. The weights of these dried and extracted samples are the dry weights; each sample should weigh approximately 0.2 mg.

#### Sample preparation for sphingolipid analysis

The method used for plant sphingolipid extraction has been described previously (Markham and Jaworski 2007; Bi et al. 2014; Zeng et al. 2021). Here we provide an overview of this method.

1. Approximately 15 mg of freeze-dried plant tissue is placed into a 2 mL Eppendorf tube to which internal standards and 1 mL of extraction solvent (hexane/water/isopropanol, 20:25:55, v/v/v) have been added. Standard A consists of C12-GlcCer, C12-Cer, and sphingosine (C17 base) dissolved at 1 µg/mL in methanol; 100 µL Standard A is sufficient for one sample. Standard B is GM1 dissolved at 1 mg/mL in tetrahydrofuran/methanol/water (2:1:2 v/v/v); 5 µL of Standard B is sufficient for one sample. The tissue is fully homogenized with an oscillatory tissue pulverizer by shaking for 2 min at 60 Hz.
2. When homogenization is complete, the sample is immediately centrifuged to ensure that both the sample and extraction solvent reach the bottom of the tube. The tube is then incubated at 60°C for 15 min.
3. Following centrifugation at 10,000 × g for 10 min, the supernatant is transferred to a new tube; the pellet is extracted once more with 500 µL of extraction solvent.
4. The samples are incubated at 60°C for 15 min and centrifuged as described above. The supernatants are combined, and 1.2 mL of the combine supernatants is divided into three 2 mL centrifuge tubes and freeze-dried under a stream of nitrogen.

### LC configurations

Extracts from plant tissues are complex and contain a variety of natural products that require further separation to obtain accurate information about lipid molecules. Many LC configurations have been described for the analysis of complex lipid mixtures. The three most important ones are reversed-phase LC (RPLC), normal-phase LC (NPLC), and hydrophilic interaction LC (HILIC). Among these methods, RPLC is the most widely used method (Cajka and Fiehn 2016). Typical methods in RPLC-based lipidomics use a short (50–150 mm) microbore column with sub-2 µm or 2.6–2.8 µm (fused-core) particle size and C18 or C8-modified sorbent (Cajka and Fiehn 2014). For a weak mobile phase, water or mixtures of organic solvents such as methanol and acetonitrile are used, while a strong mobile phase primarily consists of isopropanol or tetrahydrofuran mixed with other solvents. To improve LC separation as well as ionization and the detection of lipids, the use of mobile-phase modifiers such as ammonium formate is highly recommended. To help the reader better understand the importance of liquid phase conditions in LC–MS analysis, we will introduce some specialized LC standards that are currently used when analyzing plant lipids.

The profiles of phospholipids and galactolipids are determined by mass spectrometry as previously described (Welti et al*.* 2002; Devaiah et al. 2006; Munnik et al., 2013). Here, we present an integrated and modified version of these methods. Briefly, approximately 1 ml of plant lipid extract is collected to analyze phospholipids (PC, PE, PA, PG, PS, PI, LysoPC, LysoPE, and LysoPG) and galactolipids (MGDG and DGDG). Plant lipid extracts in CHCl_3_ are combined with solvent containing 10 μL internal standards so that the ratio of CHCl_3_/MeOH/50 mM ammonium acetate in water is 300/665/35 (v/v/v), and the final volume is 1 mL. The internal standards are acquired and quantified as previously described (Welti et al*.* 2002). Phospholipids and galactolipids are usually separated through a C18 reverse phase chromatography column. Positive and negative scan modes are used to detect all phospholipids, while galactolipids are detected in positive mode. For positive mode, mobile phase solvent A comprises methanol/acetonitrile/water at a ratio of 42.5/42.5/15 (v/v/v) with 0.1% formic acid. For mobile phase solvent B, isopropanol with 0.1% formic acid is used. For negative mode, mobile phase solvent A includes methanol/acetonitrile/water at a 42.5/42.5/15 (v/v/v) ratio along with 3 mM ammonium acetate, while mobile phase solvent B is prepared by mixing isopropanol with 3 mM ammonium acetate. The gradient elution conditions are 0–40% B at 0–10 min, 40–65% B at 10–35 min, 65–0% B at 35–42 min, and 100% A at 42–48 min for re-equilibration.

The mobile phase could be set at different starting and ending percentages to analyze sub-classes of plant sphingolipids (LCB, Cer, hCer, GlcCer, and GIPC) due to their different amphiphilic natures (Markham and Jaworski 2007; Zeng et al. 2021). Different gradients have been developed for use with various instruments. To detect LCB, Cer, hCer, and GlcCer, mobile phase solvent A is 0.2% formic acid and 2 mM ammonium formate in water, and mobile phase solvent B is 0.2% formic acid and 1 mM ammonium formate in methanol. The gradient elution conditions for LCB, Cer, hCer, and GlcCer are 80% B at 0–4 min, 80–90% B at 4–10 min, 90–99% B at 10–20 min, and 99% B at 20–26 min, with 80% B at 26–31 min for re-equilibration. To detect GIPC, the HPLC system is fitted with a fast wash station with two mixed wash solvents: wash 1, THF/methanol/water (7:2:1 v/v/v) with 0.5 mM ammonium formate, 0.1% formic acid; and wash 2, THF/methanol/water (3:2:5 v/v/v) with 2 mM ammonium formate, 0.1% formic acid. The gradient elution conditions for GIPC are 55% wash 2 at 0–0.1 min, 55–30% wash 2 at 0.1–10 min, 30–55% wash 2 at 10–10.1 min, and 55% wash 2 at 10.1–15 min.

## Lipidomics analysis tools

Three scan modes are commonly used in MS-based quantitative lipidomics: Data-Dependent Acquisition (DDA), Data-Independent Acquisition (DIA), and MRM. Unlike data from MRM, which is used for targeted lipidomics, the data from DDA and DIA are complicated and difficult to analyze. Unlike genes or proteins, metabolites are more diverse in structure, making their fragment forms very different, so de novo analysis is unavailable. Accurate quantitative lipidomics requires: (1) a professional database; (2) a theoretical match of an MS/MS spectrum; (3) an accurate match of MS/MS spectra and a chromatogram; (4) multi-dimension downstream data filtering. Data analysis is one of the most important processes in lipidomics, with a significant influence on the final results. Lipidomics detection generates a large amount of raw data, posing challenges for subsequent analysis. To meet the demands of lipidomics data processing, numerous software packages and data processing methods have been developed (Table 1). Among these tools, we highlight some common lipidomics data analysis tools below, such as XCMS, MetaboAnalyst, and LipidIMMS Analyzer.

**Table 1:** Tools employed for lipidomics

Software	Brief description	Equipment	Raw data processing	Peak annotation and data statistics		Ion mobility		MS imaging	Plant-Specific library	Language	References	Total number of citations
XCMS	An open-source R package for processing LC–MS raw data. The code can be modified to adjust to actual experimental conditions by users, but this requires a high level of computational expertise	LC–MS, GC–MS	√		√	√	×		×	R	Smith et al. 2006; Domingo-Almenara et al. 2018	3481
XCMS Online	The online version of XCMS; user-friendly but not adjustable; not able to perform many advanced types of analysis	LC–MS, GC–MS	√		√	×	×		×	R	Tautenhahn et al. 2012	910
MetaboAnalyst	A web-based free platform that offers a variety of analysis modules and workflows; analyzes all types of data (e.g. raw data, MS peaks, and annotated features)	LC–MS, GC–MS, NMR	√		√	×	×		×	Java, R	Xia et al. 2009; Xia et al. 2012; Xia et al. 2015; Chong et al. 2018; Pang et al. 2021	9591
MS-DIAL	A user-friendly graphical interface-based metabolomics analysis tool containing an untargeted real lipidome atlas	LC–MS, GC–MS	√		√	√	×		×	C#	Tsugawa et al. 2015; Tsugawa et al. 2020	1819
MZmine 3	An open-source community-driven software tool that generates vendor-specific formats for use in various downstream software platforms	LC–MS, GC–MS	√		√	√	√		×	Java	Katajamaa et al. 2006; Pluskal et al. 2010; Schmid1 et al. 2023	3118
LipiDex	A user-friendly lipidomics-specific tool that identifies isomers using its unique algorithm and generates a customized spectrum library based on the user’s needs	LC–MS	√		√	×	×		×	Java	Hutchins et al. 2018; Hutchins et al. 2019	126
LipidIMMS Analyzer	A web-based platform for lipid identification providing a four-dimension (includes ion mobility) lipidome library for the first time	LC–MS	×		√	√	×		×	R	Zhou et al. 2019	47
MetDNA	Powerful software for untargeted metabolomics that can annotate neighboring metabolites across a pathway based on seed metabolites	LC–MS	×		√	×	×		×	R	Shen et al. 2019; Zhou et al. 2022b	184
QPMASS	A powerful software tool that implements an advanced parallel algorithm, allowing 1000 samples to be processed within 17 h on a personal computer	GC–MS	×		√	×	×		×	C + +	Duan et al. 2020	13
PlantMetSuite	A web-based platform for plant metabolite identification allowing integrative multi-omics investigation and the visualization of metabolite distribution in Arabidopsis	LC–MS	×		√	×	×		√	R	Liu et al. 2023a	1
LipidSearch	A commercial search engine for LC–MS-based lipidomics that provides a large-scale lipidome database	LC–MS	√		√	×	×		×	C + +	Taguchi and Ishikawa 2010	163
LipidBlast	A specific lipid database for LC–MS that provides over 212,516 spectra, most of which were acquired in silico. It also serves as a template for unknown compounds for predicting spectra	LC–MS	-		-	-	-		-	-	Kind et al. 2013	641

### XCMS

XCMS, the acronym for various forms (X) of chromatography mass spectrometry, is the name of an open-source R package for metabolomics data analysis (Smith et al. 2006). XCMS is landmark software in the field of metabolomics. XCMS is mainly focused on LC–MS data, but GC–MS data are also allowed, and an online version for data visualization is available (https://xcmsonline.scripps.edu/). The results from XCMS can be combined with other R packages for deep data mining. For example, the results can be combined with the Mumu package to perform PCA and multivariable statistics, and they can also be combined with the ggplot2 and pheatmap packages to obtain visualization graphs. Based on the METLIN database, which provides the transitions of over 15,500 metabolites, a software package called XCMS-MRM was developed for MRM research (Domingo-Almenara et al. 2018).

The data formats used for XCMS includes netCDF, mzML, mzXML, and mzData. Raw data must be converted to one of these formats using software such as proteowizard. In brief, the first step of the XCMS workflow is to extract the peaks and correct the retention times of the same peaks from different runs. Next, XCMS will fill in the missing peaks and group them together. After the data from different samples are integrated, quantification can be performed. Although quantification is based on peak intensity, XCMS is not able to normalize the intensities of different samples, making the quantification results imperfect; this needs to be upgraded in the future. Overall, XCMS, representing the earliest open-source package for metabolomics data analysis, is powerful and widely used, and the code can be modified based on the specific demands of users.

### MS-DIAL

Mass spectrometry-data independent analysis software (MS-DIAL) is a free software package for visualized metabolomics analysis (Tsugawa et al. 2015). MS-DIAL can process various types of data from different sources, such as DDA and DIA data from GC–MS or LC–MS, and resolve metabolite structures obtained through stable isotope labeling. In June 2016, the MS-DIAL development team released version 4.0 (Tsugawa et al. 2020); the non-targeted lipidomics platform was equipped with this version of the software (http://prime.psc.riken.jp/). Version 4.0 has updated support for ion mobility information and enhanced decision tree annotation capabilities. All relevant analytical procedures are in line with the Lipidomics Standard Initiative (LSI). Moreover, the development team generated a lipid database of actual experimental data for 8,051 lipids in 117 subclasses from 1,056 sample runs using 10 types of LC–MS equipment in 81 research studies, such as retention time (RT), collision cross section (CCS), mass charge ratio (*m/z*), isotope ion, adduct type, and MS/MS. This is currently the largest lipid database with actual experimental information. Analysis of lipid annotation and semi-quantitative analysis using this platform exhibited a false positive rate of only 1–2%.

The data formats supported by MS-DIAL include ABF, mzML, netCDF, and IBF. Under certain settings, the detailed parameters can be adjusted based on specific equipment conditions to achieve optimal identification. The major parameters include adduct type, RT tolerance, *m/z* tolerance, and so on. If necessary, data filtering and quantification using an internal isotope standard can be performed in subsequent steps.

### Metabo analyst

MetaboAnalyst is a widely used online platform designed for metabolomics analysis. This platform provides a streamlined workflow suitable for researchers at all levels, from novices to senior scholars. MetaboAnalyst can process data from diverse sources, ranging from GC–MS and LC–MS to NMR data. From data normalization, batch effect removal, and functional analysis to exploratory statistics, users can perform data deconvolution to obtain the desired outputs (e.g. heatmaps, KEGG pathways, volcano plots).

In the latest version, MetaboAnalyst 5.0, numerous features have been upgraded, and the underlying codebase has been refactored to improve performance (Pang et al. 2021). Notably, raw data are allowed in version 5.0 to allow users to conduct their entire analysis in MetaboAnalyst, which avoids the need to perform raw data processing tasks with other tools using complex code commands. Moreover, MetaboAnalyst 5.0 allows other types of input data, such as MS peaks, annotated features, and generic features. For lipidomics, MetaboAnalyst 5.0 implemented a smart name-matching algorithm and expanded its lipid database by adding 197,854 lipids from RefMet and LIPID MAPS. Multi-omics analysis is another new feature, including support for data-driven analysis by the well-established DSPC (debiased sparse partial correlation) algorithm.

### Other tools

MZmine is a relatively early, powerful open-source metabolomics analysis software tool first published in 2006. In 2023, the development team released the latest version, upgrading various features (Schmid et al. 2023). MZmine 3.0 allows ion mobility data to be used in metabolite identification, resulting in more precise results. In the latest version, MS imaging is also available, offering opportunities for spatial metabolomics. Additionally, the analysis results can be exported in different formats, allowing users to easily combine them with other external tools to perform additional downstream analysis.

LipidBlast (http://fiehnlab.ucdavis.edu/projects/LipidBlast/) is an opensource lipidomics database that includes 212,516 spectra covering 119,200 lipids in 26 classes, such as phospholipids, glycerides, bacterial lipopolysaccharides, plant glycolipids, and so on. The structures and spectra of many complex glycolipids were published for the first time in this database (Kind et al. 2013). Most information in LipidBlast has been predicted in silico but has been verified using 40 types of mass spectrometry.

LipidIMMS Analyzer (http://imms.zhulab.cn/LipidIMMS/) is an online website for lipidomics analysis (Zhou et al. 2019) that was provided by the Zhu Lab from Shanghai Institute of Organic Chemistry, Chinese Academy of Sciences (CAS). Ion mobility was applied for the first time for lipid identification on this platform. A four-dimension library containing over 260,000 lipids is available on the website. The ion mobility application in MS-DIAL4 mentioned above also comes from the Zhu Lab.

PlantMetSuite is an online analysis platform specifically designed for plant metabolomics (Liu et al. 2023a). This platform allows user to annotate their raw data in over 20 plant species, perform upstream-to-downstream analysis, and plot the usual types of figures. PlantMetSuite also supports integrative multi-omics investigation. Most notably, it has a visualization database that allows users to visualize the distribution patterns of specific metabolites in different Arabidopsis tissues.

## Challenges and perspectives

**Fig. 4:**
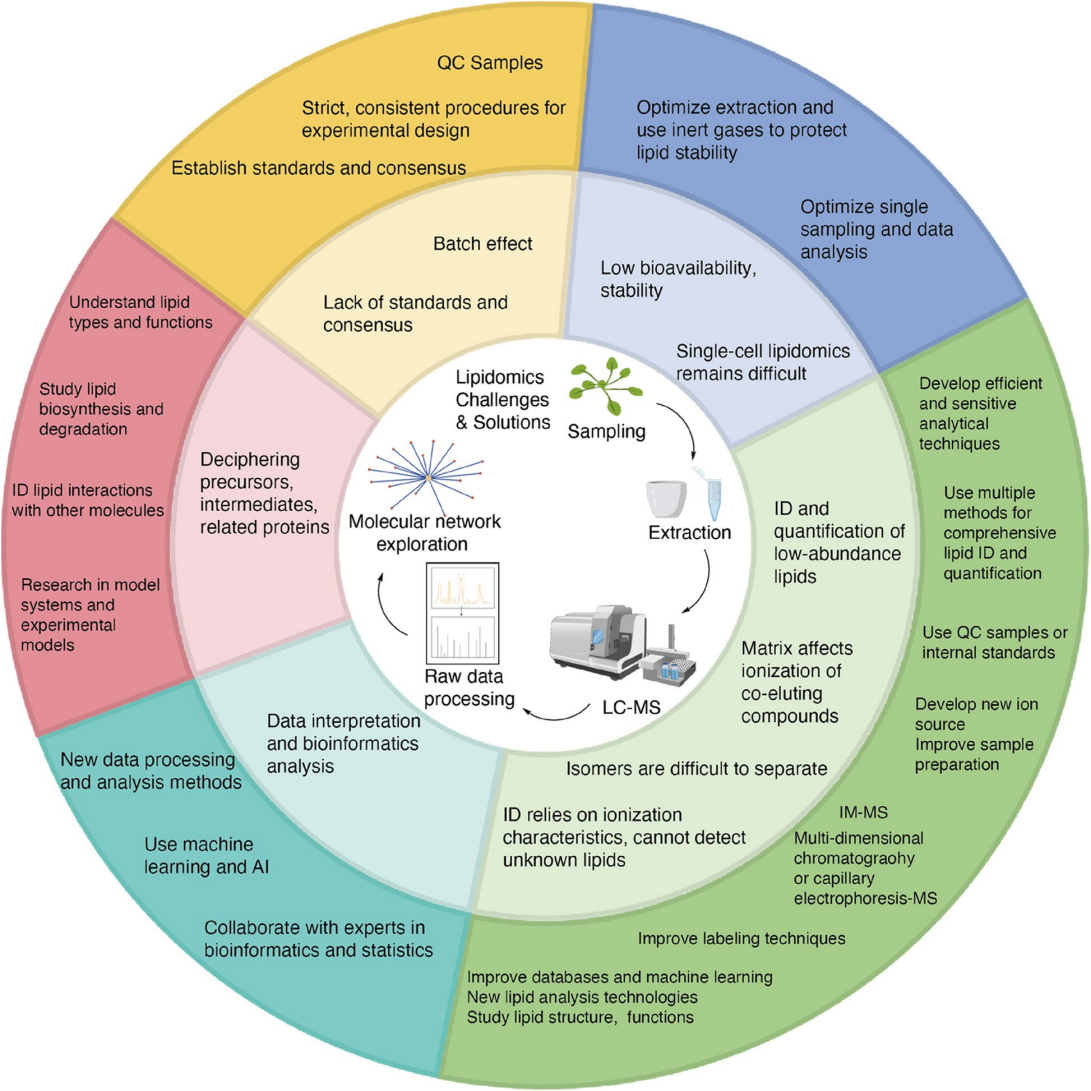
Current challenges in lipidomics and potential solutions. The challenges of lipidomics (inner circle) at various stages along with possible solutions (outer circle). For challenges in sampling and extraction, the use of inert gas for lipid extraction can improve bioavailability, which can be limited due to instability, susceptibility to oxidation, and poor water solubility (Züllig et al. 2020). For challenges in LC–MS, methods such as computational lipidomics and machine learning can be used to address challenges such as ion characteristics information dependence and data interpretation in lipidomics (Peyraud et al. 2017; Colantonio et al. 2022; Shen et al. 2023). Using labeling, isotope labeling or IM-MS techniques can distinguish isomers (Zhou et al. 2019). Ion suppression impairs the accuracy of quantification and identification by LC–MS, especially for low-abundance lipids (Annesley 2003). Researchers can use internal standards or control samples to reduce the impact of ion suppression. Quality control of samples can reduce the batch effect (Sanchez-Illana et al. 2018; Alseekh et al. 2021). Reproducibility, reusability, and transparency of data are vital concerns of lipidomics and other omics integration (Shen et al. 2023). Single-cell lipidomics requires high coverage, accuracy and advanced data analysis methods (Wang et al. 2023). It is important to establish standards and consensus for the standardization of lipidomics, the nontargeted lipidome atlas MS-DIAL 4 offers a one-stop solution for lipidome data standardization (Tsugawa et al. 2020; Shen et al. 2023)

The ultimate goals of lipidomics are to foster collaboration among existing lipidomics research centers, integrate lipid databases worldwide to standardize lipidomic analyses, and link technological advances to critical scientific issues. However, despite remarkable progress in lipidomics technologies, many challenges in plant lipidome cannot be ignored, such as challenges in data interpretation. Here we summarize the current problems faced by scientists and propose possible solutions (Fig. 4).

### Lipidomics using mass spectrometry methods

Due to the development of LC–MS and GC–MS techniques, numerous lipids have been identified and quantified. However, there are still many problems with these methods: (1) These methods are information dependent. Most LC–MS methods rely on information about the ionization characteristics of compounds and cannot detect unknown lipids. To solve this problem, researchers can improve lipid analysis by utilizing lipid databases, machine learning algorithms, and conjuncture with computational lipidomics to classify unknown lipids, although this approach requires substantial data and computational resources and enhanced accuracy and efficiency (Colantonio et al. 2022; Shen et al. 2023). Moreover, research on the structures and functions of lipids has revealed their roles and mechanisms, which could help spur additional lipid analysis and the discovery of new lipids. (2) Isomers are rarely separated under identical column conditions and are difficult to identify. The use of ion mobility can help solve this problem (Zhou et al. 2019), but high-performance ion mobility MS is still unaffordable for many laboratories. It is also possible to isolate and identify isomers more precisely using multi-dimensional chromatography or capillary electrophoresis-mass spectrometry or using labeling or isotope labeling techniques to distinguish isomers, such as lipid molecules labeled with specific isotopes, and determine the proportions of the isomers by mass spectrometry. (3) The batch effect. Unveiling the multiple of mechanism employed in organisms takes a couple of weeks or longer in large-scale studies. However, the performance of MS and sampling conditions can vary at different times, causing a batch effect that can interfere with a study. Quality control of samples can reduce this problem to a certain extent (Sanchez-Illana et al. 2018; Alseekh et al*.* 2021). (4) Ion suppression, a widely existing problem in LC–MS analysis, is caused by the matrix effect, which affects the ionization of co-eluted analytes. Ion suppression is extremely harmful to the accuracy of quantification and identification by LC–MS, especially for low-abundance lipids (Annesley 2003). Researchers can use internal standards or control samples to reduce the impact of ion suppression and can also select the appropriate analytical methods and techniques, including techniques to improve the ion source and sample handling.

### Identification and quantification of low-abundance lipids

The extremely complex structures of plant lipids make fully analyzing all lipids a formidable challenge, especially for low-abundance lipids. Extracting lipids from complex biological systems by phase separation is usually the first step in lipid analysis. After extraction, the proteins and some minerals are removed; however, the sample might not be simple enough for low-abundance lipid analysis because some substances of similar polarity remain and may affect the ionization of target molecules (Saini et al. 2021). Strategies such as optimizing extraction conditions and utilizing inert gases during lipid extraction and separation processes can mitigate alterations or losses of lipids, ensuring molecular stability (Zullig et al. 2020). Another concern is that the extract solvents could induce the hydrolysis of endogenous lipids, resulting in the artificial generation or degradation of lipids (Pati et al. 2016). Thus, methods with high selectivity must be developed for the extraction of specific lipids and the separation of different lipid molecules, which are likely to be achieved by liquid chromatography or using an updated mass analyzer.

### Standard workflow of lipidomics and consensus among laboratories

Compared to other omics fields like proteomics and genomics, lipidomics faces a notable lack of standard workflows and consensus among laboratories. This absence of uniformity poses a significant challenge when comparing and integrating findings from various research groups. Therefore, there is an urgent need to establish standards and a consensus within the realm of lipidomics. This could be achieved through the development of uniform experimental procedures and data processing methods. In 2021, Alseekh et al*.* made recommendations for standard processing methods in metabolomics and advocated a simplified report form (Alseekh et al*.* 2021). The recently created nontargeted lipidome atlas MS-DIAL 4 could offer a one-stop solution for lipidome data standardization (Tsugawa et al. 2020). By implementing such standards, we could ensure that results obtained from different laboratories are comparable, fostering collaboration and the advancement of lipidomic research as a whole. Standardization efforts should encompass various aspects of lipidomics, including sample preparation, mass spectrometry techniques, data analysis, and reporting guidelines. This will not only enhance the quality and reliability of lipidomic data but also enable the scientific community to harness the full potential of lipidomics for understanding complex biological systems. Furthermore, the establishment of standardized reference materials and analytical platforms can aid in the validation and harmonization of results, ultimately advancing our knowledge of lipids.

### Unexplored biological mechanisms of plant lipids

It is well known that lipids are widely involved in various signaling pathways, but the details are currently unclear. For example, our understanding of the sphingolipid mechanism is still in its rudimentary stages. In the past decade, several studies have shown that sphingolipids undergo crosstalk with almost all plant hormones (Wu et al. 2015; Corbacho et al. 2018; Yang et al. 2019; Huang et al. 2021; Zeng et al. 2021). However, how sphingolipids interact with other signaling molecules remains a mystery. Apart from signaling pathways in plants, the patterns of sphingolipids are also intricate in both mammals and plants. After being treated with Fumonisin B1 (FB1), a water-soluble toxic from *Fusarium verticillioides*, C16 Cer levels decreased in mammals but increased in plants (Markham et al. 2011; Aflaki et al. 2012; Zeng et al. 2020), highlighting the complexity of sphingolipid-modulated mechanism. Even though more than 200 species of sphingolipids have been identified to date (Cahoon et al. 2021), the plant cellular sphingolipidome comprises at least 500 different species of sphingolipids (Pata et al. 2010). More target proteins of sphingolipids and their underlying mechanism need to be explored in the future; such studies will be facilitated by multi-omics analysis.

### Benchmarked joint multi-omics analysis

Association analysis of lipidomic data with genomics, transcriptomics, and proteomics data provides a powerful way to explore physiological mechanisms. A method combining lipidomics and genomics called metabolome genome-wide association study (mGWAS) was designed to explore the relationships between metabolites and genes. A recent mGWAS of 52 lipid-related metabolites with 214 soybean (*Glycine max*) accessions led to the generation of a three-dimensional genetic network of phenotypes, metabolites, and genes (Liu et al. 2020a). Association analysis of lipidomics data with transcriptomics and proteomics data is often used to explore pathways. For instance, the biosynthetic pathway of steroidal glycoalkaloids in potato (*Solanum tuberosum*) and tomato (*S. lycopersicum*) was revealed through association analysis of transcriptomics and metabolomics data (Itkin et al. 2013). Moreover, in an association analysis of proteomics and lipidomics based on the Arabidopsis autophagy mutant *atg5*, the levels of peroxisome-related proteins, ER-related proteins, phospholipids, and sphingolipids were shown to change dramatically in the mutant, indicating that autophagy is extremely important for ER stress, lipid homeostasis, and inner membrane composition (Have et al. 2019). Traditionally, researchers have preferred to explore certain lipids or proteins that show dramatic changes in levels (Raffaele et al. 2008), but this is not sufficient due to the existence of redundant components. Model construction, a machine learning method, has gradually begun to play a major role in multi-omics analysis, as it can predict phenotypes and unveil the interactions of biological molecules (Peyraud et al. 2017). However, reproducibility, reusability, and transparency could become crucial issues due to the massive amounts of data generated daily in multi-omics studies. Integrating lipidomics and other omics datasets must become more systematic in order to understand complex biological networks and build a more thorough biological knowledge base by addressing these issues (Shen et al. 2023).

### Imaging spatiotemporal changes in plant lipids

The visualization of lipids is an important way to explore their functions in various biological processes. A universally implemented method to image plant lipids is to employ genetically encoded biosensors based on fluorescence and lipid–protein interactions (Vermeer and Munnik 2013; Walia et al. 2018). Perhaps the greatest drawback associated with the use of lipid biosensors is that they can sequester their target lipids and hence disrupt physiological interactions with effector proteins. Another key point is that the biosensor is expressed in the cell independently of the presence of its lipid target. Therefore, changes in local concentrations of the lipid alter the localization of the biosensor and not its overall expression level or total fluorescence. These issues preclude the use of this technique for low-abundance plant lipids. The development of Mass Spectrometry Imaging (MSI) provided a new opportunity for lipidomics visualization. This method uses MALDI to scan the sample surface, mix it with a matrix, and apply it to a metal plate. The lipid molecule and matrix undergo desorption and ionization, and the signal intensity of the ions is examined by MS. The resulting data can be processed using image processing software to reveal the distribution and proportion of a certain lipid in different areas, making the visualization of lipids possible (Klein et al. 2018). This technique provides a convenient way to explore the distribution of lipids in plant cells. Lipotype classification in human fibrocytes was successfully performed in a recent study using MADLI-MSI and single-cell lipidomics analysis (Capolupo et al*.* 2022). Even though MALDI-MSI has achieved some exciting results, this method still has some drawbacks, such as weak performance in terms of scan range and resolution, matrix limits, and difficulty in comparing different samples (Li et al. 2014; Bartels and Dörmann 2021; Pathmasiri et al. 2021).

### Development of single-cell lipidomics

Classical lipidomics provides a map of the average cell population, reflecting its general biological state, but ignores the spatial distribution of lipids, which is often important in biology. The important role of sphingolipids in determining the state of fibroblasts was revealed through the detection of lipid compositions in individual cells, further confirming that intercellular heterogeneous lipid metabolism plays a role in guiding the self-organization of multicellular systems (Capolupo et al*.* 2022). Therefore, single-cell lipidomics is also an important future direction. However, to date, little research on single-cell lipidomics in plants has been performed. The ultimate goal of single-cell lipidomics is to create accurate maps of single-cell lipidomes to reveal subtle differences between cells. Changes in lipid levels in single cells are small compared to changes at the transcriptome level. Thus, high coverage, accurate identification, and quantitative measurements are needed to accurately interpret these subtle but meaningful changes. The creation of a lipid tag library or analysis using a pool of cells of a single type (Misra et al*.* 2014) may make it easier to address this problem. In addition, the analysis of omics data is essential for gaining useful biological insights. However, single-cell lipidomics is at an early stage of development, and no systematic data analysis system has thus far been established. Single-cell data sets are nosier and more sparse than standard data sets, which exacerbates the challenge of single-cell lipidomics data analysis (Wang et al*.* 2023). Given the current limitations in lipid coverage in single-cell lipidomics, long-term efforts are needed to resolve the bottleneck of data analysis in single-cell lipidomics.

As a whole, high-throughput and high-accuracy lipidomics techniques must promote the development of lipidomics. At the same time, single-cell lipidomics analysis using a combination of targeted/untargeted lipidomics and visualized lipidomics will provide a new viewpoint for refined lipidomics studies. The application of lipidomics techniques has allowed for the identification and quantification of a wide range of lipids, providing insights into their roles in plant metabolism, signaling, and responses to environmental stress. Such information should ultimately lead to the development of more sustainable, efficient agricultural practices and the creation of novel plant-based products.

## Data Availability

No datasets were generated or analyzed during the current study.
